# An analytical approach to reduce between-plate variation in multiplex assays that measure antibodies to *Plasmodium falciparum* antigens

**DOI:** 10.1186/s12936-017-1933-6

**Published:** 2017-07-17

**Authors:** Rui Fang, Andrew Wey, Naveen K. Bobbili, Rose F. G. Leke, Diane Wallace Taylor, John J. Chen

**Affiliations:** 1 Office of Biostatistics and Quantitative Health Sciences, University of Hawaii John A. Burns School of Medicine, Honolulu, HI 96813 USA; 2 Department of Tropical Medicine, Medical Microbiology and Pharmacology, University of Hawaii John A. Burns School of Medicine, 651 Ilalo Street, BSB 211, Honolulu, HI 96813 USA; 3 Biotechnology Center, Faculty of Medicine and Biomedical Research, University of Yaounde 1, Yaounde, Cameroon

**Keywords:** Antibodies, Between-plate variation, Multiplex assay, Normalization procedure, Malaria, Placental malaria

## Abstract

**Background:**

Antibodies play an important role in immunity to malaria. Recent studies show that antibodies to multiple antigens, as well as, the overall breadth of the response are associated with protection from malaria. Yet, the variability and reliability of antibody measurements against a combination of malarial antigens using multiplex assays have not been well characterized.

**Methods:**

A normalization procedure for reducing between-plate variation using replicates of pooled positive and negative controls was investigated. Sixty test samples (30 from malaria-positive and 30 malaria-negative individuals), together with five pooled positive-controls and two pooled negative-controls, were screened for antibody levels to 9 malarial antigens, including merozoite antigens (AMA1, EBA175, MSP1, MSP2, MSP3, MSP11, Pf41), sporozoite CSP, and pregnancy-associated VAR2CSA. The antibody levels were measured in triplicate on each of 3 plates, and the experiments were replicated on two different days by the same technician. The performance of the proposed normalization procedure was evaluated with the pooled controls for the test samples on both the linear and natural-log scales.

**Results:**

Compared with data on the linear scale, the natural-log transformed data were less skewed and reduced the mean–variance relationship. The proposed normalization procedure using pooled controls on the natural-log scale significantly reduced between-plate variation.

**Conclusions:**

For malaria-related research that measure antibodies to multiple antigens with multiplex assays, the natural-log transformation is recommended for data analysis and use of the normalization procedure with multiple pooled controls can improve the precision of antibody measurements.

**Electronic supplementary material:**

The online version of this article (doi:10.1186/s12936-017-1933-6) contains supplementary material, which is available to authorized users.

## Background

Antibodies play an important role in immunity against *Plasmodium falciparum* infections. Studies show that antibodies to a combination of antigens, not just a single antigen, are associated with protection from malaria [1–5]. Although the precise combination of antigens has not been identified, future studies will need to measure antibody levels against multiple malarial antigens as the breadth of the antibody response has been associated with protection [6, 7] and antibodies to different antigens can be used to estimate if a person has been infected within the last 30, 90 or 365 days [8].

Traditionally, antibodies to malarial antigens have been measured using an enzyme-linked immunosorbent assay (ELISA) that measures antibody to only one antigen at a time. However, recently, multiplex bead-based assays have been developed for measuring antibodies to multiple antigens simultaneously in the same well [9–12], thereby, reducing cost, time, as well as the amount of plasma and antigen. Although routinely used today, the variability of multiplex assays for malarial antigens has not been well characterized. Other studies have shown that measurement variability in multiplex assays depends on the substance being measured [13, 14], making it important to evaluate measurement variability of antibodies to different malarial antigens.

For multiplex assays, measurement error occurs both within- and between-plates [15]. Within-plate variability is the variation among replicates of the same sample for an antigen in different wells on the same plate. Between-plate variability is the variation that occurs from running the same sample on different plates. The within-plate variability is inherent to the assay, e.g., variation caused by pipetting and mixing. In contrast, between-plate variability can be reduced during the study design stage and/or through appropriate statistical adjustments at the data analysis stage [16].

Several general approaches for data normalization have been reported. For example, logarithmic transformations have been used to reduce the skewness of the data and alleviate the association between the variance and mean of the responses, i.e., the mean–variance relationship [16, 17]. A loess-based normalization procedure has been developed for RNA expression data [18] with application to multiplex assays in transplantation studies [19]. The loess-based normalization procedure assumes that the distributions of gene expression levels are rank invariant, i.e., the same rank order of expression levels across the samples for different genes [20]. Yet this rank invariant assumption may not held for different malarial antigens of plasma samples, since multiple factors influence a person’s B cell response to malaria. Quantile normalization is commonly used in gene expression data by implementing a transformation such that each plate has the same distribution [20]. Both LOESS and quantile normalizations are intended to globally normalize across different genes/antigens. This general approach is different from the goal of the current study which is to reduce between-plate variations for each antigen separately. While ranks should be invariant for the controls, which are included on every plate, the invariant assumption may be untenable for the test samples which are likely to be assayed only once in malaria research. Analysis of variance (ANOVA)- and regression-based normalization procedures are also commonly used in proteomics [21, 22] and multiplex assays outside of malaria research. However, the effectiveness of ANOVA-based normalization procedures depends on the substance being measured [13, 14], such as types of Human Leukocyte Antigens [23]. Thus, extending ANOVA-based normalization procedures to malaria-specific antigens requires validation.

The objective of the study was to reduce between-plate variability associated with measuring antibodies to a combination of malarial antigens based on multiplex assays at the data analysis stage. Positive pooled controls were developed based on the average ranks of malaria antigens of interest, using archival plasma samples. A simple ANOVA-based normalization procedure was tested that uses between-plate replicates of pooled controls to normalize test samples across different plates on both the linear and natural-log scales. First the differences between the observed and the averaged values for the pooled controls across plates (i.e., the estimated plate effects) were calculated, and then test samples were adjusted using the estimated plate effects. The performance of the proposed normalization procedure was then evaluated by comparing the between-plate variation with versus without the normalization.

## Methods

### Malarial antigens

A multiplex bead-based immunoassays was used to measure antibody levels to nine malarial antigens, including merozoite antigens, sporozoite CSP, and pregnancy-associated VAR2CSA. These nine antigens include recombinant proteins, gene expression proteins, and synthetic peptides. Recombinant proteins included: Apical merozoite protein 1 (AMA1) 3D7 strain expressed in yeast (molecular weight 83 kD), the c-terminal 42,000 portion of merozoite surface protein-1 (MSP1-42) expressed in *Escherichia coli*, kindly provided by C. Long Malaria Vaccine Development Branch (MVDB), NIAID, NIH; MSP2 (Fc27 strain) expressed in yeast, kindly provided by R. Anders (LaTrobe University, Australia); MSP3 (HB3) expressed in *Escherichia coli*, kindly provided by J. Rayner (Wellcome Sanger Institute, UK); Erythrocyte binding antigen-175 RII (EBA-175), obtained from Science Applications International Corp., Frederick, MD; and full-length VAR2CSA (FV2) (FCR3) expressed in Baculovirus from A. Salanti (University of Copenhagen, Denmark). The extracellular domains of full length MSP11 and Pf41 (merozoite surface antigens) were made by gene synthesis and expressed as biotinylated proteins in MEK293E cells [24], provided by Rayner. Finally, a synthetic peptide consisting of five PNAN repeats (molecular weight 2.1 kD) coupled to bovine serum albumin (BSA) via an added terminal cysteine residue (CPNANPNANPNANPNANPNAN), was synthesized by AnaSpec, Inc. (San Jose, CA). Detailed information on the antigens is provided in Additional file 1: Table S1.

### The multi-analyte platform (MAP) assay

The MAP assay was performed as previously described [25]. Recombinant and synthetic proteins were directly coupled to SeroMAP microspheres (Luminex Corp., Austin, TX) [16]; whereas, for the gene expression proteins, one million SeroMAP microspheres were covalently coupled with 50 µg of Streptavidin (Pierce Biotechnology, Rockford, IL) and then the microspheres were incubated in antigen-containing supernatants at saturating levels. After coupling, the antigen-coupled microspheres were combined in equal numbers to create a 9-plex mixture. In the assay, 50 µl of the 9-plex mixture containing 2000 microspheres of each antigen was combined with 50 µl of plasma diluted to 1:200 using phosphate buffered saline (PBS) containing 1% BSA, in wells of pre-wetted filter plates (96 well Multiscreen BV; Millipore, Billerica, MA) and incubated for 1 h and 30 min at 25 °C on a rotating shaker at 500 rpm (Microplate Shaker, Lab-line, Melrose Park, IL). Microspheres were washed twice with PBS-0.05% Tween20 and once with PBS-1% BSA. Then, 100 µl of secondary antibody (R-phycoerythrin-conjugated, Affini Pure F(ab’)_2_ fragment, Goat anti-human IgG Fc fragment specific, Jackson Immunoresearch, West Grove, PA, Cat # 109-116-170) diluted to 2 µg/ml in PBS-1% BSA was added to each well and incubated as described above in the dark for 1 h. Wells were washed, microspheres were re-suspended in 100 µl PBS-1% BSA and 85 µl of the microsphere suspension was analysed using a Liquichip M100 reader (Qiagen, Valencia, CA). The reader was programmed to read a minimum of 100 beads per spectral address, DD Gate 7500–15,000 and 35 s timeout. The results were expressed as median fluorescent intensity (MFI).

### Preparation of positive and negative controls

In a prior study, archival, coded plasma samples from 1377 pregnant women residing in Yaoundé, Cameroon, a malaria-endemic area, were screened for antibodies to 28 malarial antigens [26]. Antibody data from that study were used to created positive serological controls in the current study. In an attempt to prepare controls containing a range of antibodies levels to each antigen, antibody levels for each of the seven antigens were ranked from high to low. Based on the average rank across the seven antigens, the samples were then evenly divided into deciles. The top 10 samples from deciles 1, 3, and 5, and the bottom 10 samples from deciles 8 and 10 were separately pooled to create five positive pooled controls (PC1–PC5) (n = 10 samples per pooled PC), with antibody levels ranging from high to low. In addition, 30 plasma samples from 30 Cameroonian adult males that lack antibodies to FV2 were pooled to create a PC for the asexual-stage antigens, but a negative control for FV2 (FV2-NC). Finally, another negative pooled control was created by pooling 30 plasma samples from pregnant women residing in the USA who had never travelled to a malaria endemic region (US-NC).

### Study design

After excluding samples included in the pooled controls, 30 malaria-positive (M+) and 30 malaria-negative (M−) plasma samples were randomly selected from the 1327 remaining samples for a total of 60 samples for testing. These 60 test samples were assayed in triplicate (i.e., three different wells on the same plate) with 20 samples (10 M+ and M−) on each of three different plates on a single day. Then, the same technician duplicated the process and reran three plates of the same samples on another day, with the same plate and well assignments as on the first day. On each of six plates, the same panel of seven pooled controls was also assayed in triplicate.

### Statistical methods

The descriptive statistics of antibody levels (expressed as MFI) were summarized for each antigen on both the linear and natural-log scales. The proposed adjustment approach to reduce between-plate variation was built on the plate effect estimated using the same group of pooled controls assayed on all plates. The key idea was that the MFI for a sample (e.g., the pooled controls) should remain constant across different plates for each antigen. The estimated plate effect for each antigen was then the average difference of the pooled controls on a given plate from the overall means of the corresponding controls across all plates. Four different sets of controls were considered to estimate the plate effect: five positive pooled controls only (PC1–PC5, n = 5); five PC plus the US-negative control (PC1–PC5 plus US-NC, n = 6); five positive controls plus the pool of Cameroonian male control (i.e., a PC for the malarial antigens but a negative control for FV2) (PC1–PC5 plus FV2-NC, n = 6); and all 7 pooled controls (n = 7). For each antigen, the proposed analytical procedure for reducing between-plate variation included the following 6 steps: (1) Obtain the linear MFI value for pooled control samples and test samples; (2) Convert the MFI values to natural-log values; (3) For each pooled control, calculate the differences between the mean MFI averaged across the replicates for each plate and the overall mean MFI values across both replicates and plates; (4) Obtain the estimated plate effect for a specific plate by averaging the calculated differences across all pooled controls; (5) For test samples on a given plate, obtain the adjusted MFI values by subtracting the estimated plate effect from the unadjusted MFI. Steps 3–5 were conducted in both linear and natural-log scales.

Specifically, let y_ijk_ be the MFI value for the ith pooled control on the jth plate and the kth replicate. Given a balanced design in which there are I controls, J plates, and K replicates, the overall mean for the ith pooled control was then $\overline{y}_{{{\text{i}}..}} = \frac{1}{{{\text{J}} \times {\text{K}}}}\sum\nolimits_{{{\text{j}} = 1}}^{\text{J}} {\sum\nolimits_{{{\text{k}} = 1}}^{\text{K}} {{\text{y}}_{\text{ijk}} } }$. For the current study, there were I controls (I = 5, 6, 7), six plates (J = 6), and three replicates (K = 3). This means that $\overline{y}_{{{\text{i}}..}} = \frac{1}{18}\sum\nolimits_{{{\text{j}} = 1}}^{ 6} {\sum\nolimits_{{{\text{k}} = 1}}^{ 3} {{\text{y}}_{\text{ijk}} } }$ and the estimated plate effect for ith pooled control was $\frac{1}{3}\sum\nolimits_{{{\text{k}} = 1}}^{ 3} {{\text{y}}_{\text{ijk}} - \overline{y}_{{{\text{i}}..}} }$. The final estimated plate effect for jth plate was then the average of the plate effects for each pooled control: $\widehat{\upbeta}_{\text{j}} = \frac{1}{\text{I}}\sum\limits_{{{\text{i}} = 1}}^{\text{I}} {\left( {\frac{1}{3}\mathop \sum \limits_{{{\text{k}} = 1}}^{3} {\text{y}}_{\text{ijk}} - \overline{\text{y}}_{{{\text{i}}..}} } \right)}$.

Note that $\widehat{\upbeta}_{\text{j}}$ was the average difference of the jth plate from the overall mean of the pooled controls. Let z_ijk_ be the unadjusted MFI value for the ith test sample, jth plate, and kth replicate. The adjusted MFI value for the test sample, $\widetilde{\text{z}}_{\text{ijk}}$, was then obtained by subtracting the estimated plate-effect ($\widehat{\upbeta}_{\text{j}}$) from the unadjusted MFI of test sample on the given plate; that is, $\widetilde{\text{z}}_{\text{ijk}} = {\text{z}}_{\text{ijk}} - \widehat{\upbeta}_{\text{j}}$. The notation was changed from y_ijk_ to z_ijk_ to emphasize that test samples were not used in the estimation of the plate effects.

For each antigen, the between-plate variation was calculated by first averaging the three within-plate replicates. The between-plate variation for the unadjusted MFI values was then estimated by averaging the squared differences of the unadjusted MFI values between the two plates for the 60 test samples. Similarly, the adjusted between-plate variations were estimated by the average squared differences of the adjusted MFI values between the two plates for the sixty test samples. The impact of the adjustment on between-plate variation for each antigen was investigated on both the linear and natural-log scales. Paired t test was used to compare between the unadjusted and adjusted values in between-plate variations. Data were analysed using the SAS software version 9.4 (SAS Institute Inc., Cary, NC, USA). A two-sided test was used and a p < 0.05 was regarded as statistically significant.

## Results

**Fig. 1:**
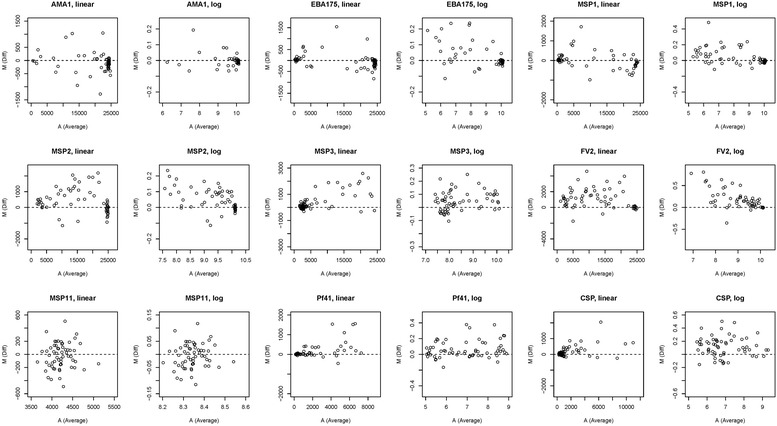
Nine pairs of MFI difference values (M) versus average MFI values (A) plots using both linear and natural-log scales

Table 1 summarizes the amount of antibodies present in the 60 test samples for each antigen on the linear and natural-log scales. On the linear scale, the MFI data were highly skewed. In contrast, the natural-log scale transformation resulted in the data being less skewed. To better understand the mean–variance relationship between linear and natural-log scales, MFI difference values (M) versus average MFI values (A) between plates from test samples were plotted (M–A plot) for each antigen (Fig. 1). The plots show that the mean–variance relationship existed in most of antigens (i.e., the differences are bigger at high MFI values) on the linear scale; whereas, the natural-log transformed reduced this relationship (i.e., the pattern was at random).

**Table 1:** Summary of MFI values for the 60 test samples on both the linear and natural-log scales

Antigen	Linear scale					Natural-log scale				
	Mean	SD	Median	Minimum	Maximum	Mean	SD	Median	Minimum	Maximum
Recombinant protein										
AMA1	19,899	7127	23,855.5	477	25,361.5	9.717	0.822	10.08	6.168	10.14
EBA175	14,807	10,410	21,146.5	132.5	24,862	8.899	1.572	9.959	4.887	10.12
MSP1	9870	9849	4599.8	188	24,474	8.183	1.713	8.434	5.236	10.11
MSP2	15,488	8406	15,558	1626.5	26,257.5	9.409	0.794	9.652	7.394	10.18
MSP3	7051	6947	3251	1426	24,323	8.453	0.860	8.087	7.263	10.10
FV2	12,073	7846	11,210.5	556	25,858.5	9.099	0.872	9.325	6.321	10.16
Gene expression protein										
MSP11	4212	320	4186.8	3389	5414	8.343	0.075	8.340	8.128	8.597
Pf41	1970	2144	1019.5	141	8297	6.878	1.270	6.927	4.949	9.024
Synthetic peptide										
CSP	1825	2365	821.3	207	12,088	6.931	1.015	6.711	5.333	9.400

**Fig. 2:**
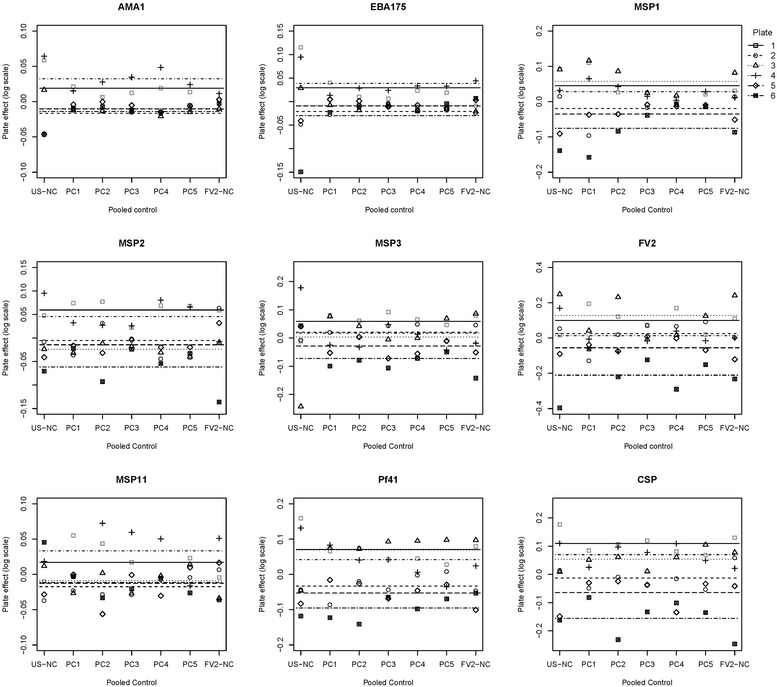
An illustration of reduction of the plate effect using data on the natural-log scale for each antigen using all seven pooled controls. *Each dot* represents the estimated plate effect of one pooled control on each of the six plates, i.e., after subtracting the overall mean of the corresponding pooled control across all six plates. *Each horizontal dotted line* represents the final estimated plate effect, which is the average across the seven pooled controls

Table 2 presents the between-plate variation for the unadjusted and adjusted MFI values for each antigen on both the linear and natural-log scales. On the linear scale, the unadjusted between-plate variance was relatively large and the different adjustments could only reduce the variation for some antigens, as indicated by adjusted-to-unadjusted ratios lower than 1.0. In contrast, the normalization procedure performed well on the natural-log transformed MFI data, i.e., adjusted-to-unadjusted ratios were much lower than those of the linear scale for most antigens. In particular, the adjustments using 6 controls (including the negative control) or using all seven pooled controls reduced the between-plate variance for all antigens. Figure 2 provides the estimated plate effects plotted on the natural-log scale for each antigen using all seven pooled controls.

**Table 2:** Reduction of between-plate variation for MFI on the linear and natural-log scales

Antigen	Linear scale					Natural-log scale				
	Unadjusted variance	Adjusted^a^ (I = 5) to unadjusted ratio	Adjusted^b^ (I = 6) to unadjusted ratio	Adjusted^c^ (I = 6) to unadjusted ratio	Adjusted^d^ (I = 7) to unadjusted ratio	Unadjusted variance	Adjusted^a^ (I = 5) to unadjusted ratio	Adjusted^b^ (I = 6) to unadjusted ratio	Adjusted^c^ (I = 6) to unadjusted ratio	Adjusted^d^ (I = 7) to unadjusted ratio
Recombinant protein										
AMA-1	139,412	1.183	1.079	1.160	1.077	0.0013	1.024	0.912	1.026	0.925
EBA175	125,497	1.013	0.966	1.269	1.151	0.0060	1.030	0.900	1.083	0.943
MSP1	167,994	0.905	0.889	0.992	0.957	0.0121	0.609	0.598	0.598	0.606
MSP2	759,821	0.928	0.927	0.873	0.863	0.0070	0.849	0.844	0.751	0.759
MSP3	676,301	0.887	0.725	0.874	0.733	0.0076	0.801	0.658	0.903	0.631
FV2	2,679,797	0.659	0.539	0.576	0.506	0.0739	0.473	0.445	0.452	0.444
Gene expression protein										
MSP11	37,028	0.842	0.875	0.811	0.850	0.0021	0.862	0.894	0.830	0.869
Pf41	170,559	0.621	0.596	0.615	0.600	0.0155	0.279	0.289	0.288	0.299
Synthetic peptide										
CSP	157,499	0.742	0.760	0.727	0.743	0.0355	0.400	0.395	0.369	0.372

The ratios assess the variance reduction of the different adjustments with values less than one indicating a reduction of between-plate compared to the unadjusted values.

^a^Adjustment using 5 pooled controls from pre-screened samples (PC1–PC5)

^b^Adjustment using 6 pooled controls including the pooled negative female control (PC1–PC5 plus US-NC)

^c^Adjustment using 6 pooled controls including the pooled negative male control (PC1–PC5 plus FV2-NC)

^d^Adjustment using all 7 pooled controls

**Fig. 3:**
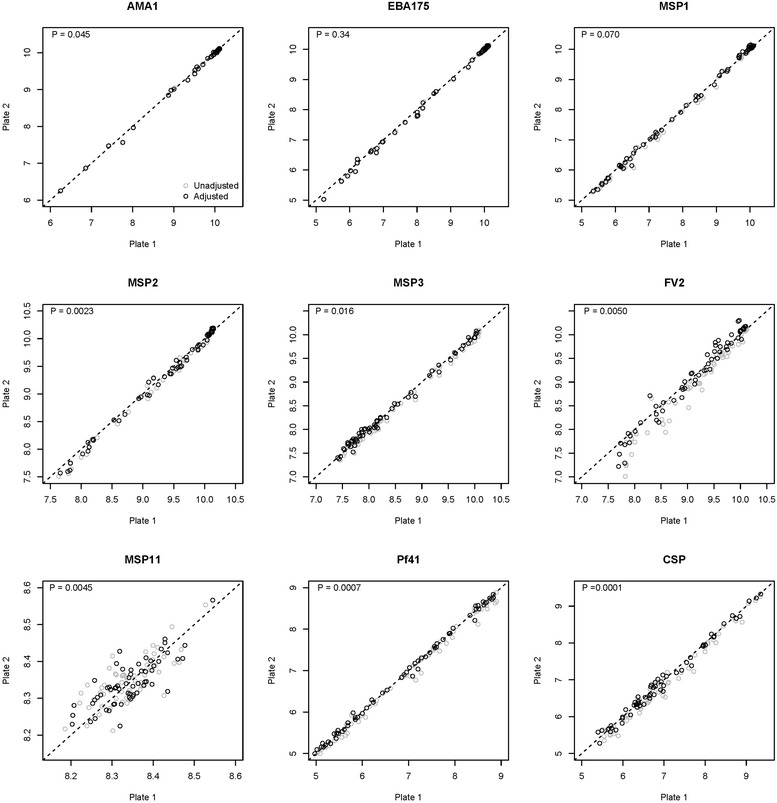
Concordance of unadjusted versus adjusted MFI values between two plates in 60 test samples for all antigens on the natural-log scale. The sample MFI values closer to the line Y = X line indicate reduction of plate-to-plate variation after the adjustment on the natural-log scale. p values were based on paired t tests, comparing between unadjusted and adjusted values in between-plate variations

Figure 3 shows the effect of the normalization procedure when the linear MFI of the test samples were plotted together with the adjusted values using all seven pooled controls on the natural-log scale for each antigen. Compared with the unadjusted values, the adjusted MFI values were significantly closer to the Y = X line for eight of the nine antigens (paired t test p < 0.05), which represents perfect concordance, i.e., no plate-to-plate variation. Thus, the adjustments on the natural-log scale demonstrated the feasibility of reducing between-plate variation through a normalization procedure using pooled controls.

## Discussion

This investigation demonstrated that the natural-log transformation of MFI data was more consistent than the linear MFI scale for malaria-related antibodies using the multiplex assay. The mean–variance relationship based on the natural-log transformed MFI values was reduced.

The proposed normalization procedure using multiple pooled controls significantly reduced the between-plate variation on the natural-log scale. Compared with other normalization procedures, a major strength of the proposed ANOVA-based normalization procedure is its simple implementation. As long as a given laboratory has a well-defined set of controls, a multiplex assay can be easily adjusted by calculating the difference between the observed controls and the average values for the controls across plates (i.e., the plate effect). In addition, despite the replication built into this study, the proposed normalization procedure does not require within- or between-plate plate replicates of the test samples. Only the pooled controls are required across plates. Finally, in contrast to other normalization methods, the proposed normalization maintains a straightforward interpretation of final study results by back-transforming the adjusted values to the linear scale. However, there exists residual plate-effect after adjustment. This indicates that the normalization procedure is not-optimal. One assumption of the proposed normalization procedure is that the pooled controls possess the same plate effects as the test samples. However, the existence of a plate-by-sample interaction where the plates effects are different for pooled controls and test samples would have violated this assumption. Testing the plate-by-sample interaction on pooled controls is a potential approach to assess the validity of the normalization procedure. Any statistical approach for assessing the validity of the normalization approach could be improved by also incorporating information on antibody characteristics that could impact the between-plate adjustments.

The selection of controls is critical for normalization. In the current study, five pooled positive controls were prepared based on the deciles of the average antibody response using pre-screened samples. The pre-screening of the pooled controls may also ensure that each control better covers the range of potential MFI values with variant ranks across antigens. By pooling different samples with different ranks of the antigens, it had hoped to achieve a range of expression levels to all antigens. The results for pooling samples as shown in Additional file 2: Table S2 suggest that this goal was not fully achieved. An alternative approach would be to pool positive controls and create a serial dilution of this pooled sample. However, it might still be challenging to control the dilutions to reflect the actual ranges for MIF values across different antigens in a multiplex assay, for example, different antigens would require different dilution ratios.

Between-plate replication of test samples was included to evaluate the feasibility of the proposed normalization procedure. However, in practice, the proposed normalization procedure only requires between-plate replication of the pooled controls and not the test samples. This should help the investigation of responses to multiple malaria antigens across many subjects as between-plate replication of test samples may not be feasible due to limited amounts of biological specimen. Yet, since the proposed normalization procedure only relies on the pooled controls, residual plate-effects may still exist for the test samples after the proposed adjustment. Regardless, the current model should be useful in most situations.

Overall, the proposed normalization procedure has significant advantages. The procedure does not assume that the between-plate distributions are rank invariant and can be easily implemented. The simplicity in the estimation of the plate effects improves the transparency of the final adjusted values of the test samples. Most importantly, the normalized MFI values result in lower between-plate variation on the natural-log scale. This should help reduce the overall variation and improve the estimation and statistical power for malaria-related research using multiplex assays.

An analytical method for data produced by multiplex assays is important since future malaria studies will require the measurement of antibodies to multiple antigens, including large scale studies that require the evaluation of samples on multiple assay plates. Results from this study provide a method for analysis of data from multiplex assays. Further studies are needed to help scientists understand the consequences of failing to control for between-plate and, more generally, between-assay variability, on the accuracy of predicting outcome based on antibodies data.

## Conclusions

The natural-log transformation should be used for data analysis in using malaria antibody measurements. The normalization procedure using pooled controls could reduce the between-plate variability and improve the precision of malaria related research using multiplex assays.

## Additional files

**Additional file 1: Table S1.** List of antigens used in the study.

**Additional file 2: Table S2.** Summary of MFI values in linear scale for the pooled control samples across six plates.

## Abbreviations

Ab: antibody/antibodies
ELISA: enzyme-linked immunosorbent assay
MFI: median fluorescent intensity
ANOVA: analysis of variance
AMA: apical merozoite protein
MSP: merozoite surface protein
EBA: erythrocyte biding antigen
FV2: full length VAR2CSA
M+: malaria-positive
IE: *P. falciparum*-infected erythrocytes
M−: malaria-negative
MAP: multi-analyte platform
PC: positive control(s)
NC: negative control(s)
