# Supplementary material for: An analytical approach to reduce between-plate variation in multiplex assays that measure antibodies to Plasmodium falciparum antigens
Source: Malar J. 2017 Jul 17;16:287. doi: 10.1186/s12936-017-1933-6 (PMC5513105; doi:10.1186/s12936-017-1933-6)
Supplement: Supplementary file 1 — Additional file 1: Table S1. List of antigens used in the study. [file 12936_2017_1933_MOESM1_ESM.doc]

Supplemental Table 1: List of Antigens Used in the Study

| Name |  | Method of Production | Source |
| --- | --- | --- | --- |
| Apical Merozoite Antigen-1 (3D7) | AMA-1 | expressed in yeast;  mw 83 kDa | Malaria Vaccine Development Branch, NIAID, NIH |
| Merozoite Surface Protein-1 c-terminal (3D7) | MSP1_42_ | *Escherichia coli*;  mw 42 kDa | Malaria Vaccine Development Branch, NIAID, NIH |
| Erythrocyte-Binding Antigen-175 region II | EBA-175 RII | expressed in yeast;  mw 60 kDa | Science Applications International Corp., Frederick, MD |
| Merozoite Surface Protein-2 | MSP2 | no N-terminal signal and c-terminal GPI-attachment sequence, but containing an N-terminal six-HIS tag | R. Anders, La Trobe University, Victoria, Australia |
| Merozoite surface Protein 3 | HB3 | *E. coli*; C-terminal region | Julian Rayner, Wellcome Trust Sanger Institute, UK |
| Merozoite Surface Protein - 11 | MSP11 | gene synthesis; expressed as biotinylated proteins in MEK293E cells- full length | Julian Rayner, Wellcome Trust Sanger Institute, UK |
| Merozoite Surface Antigen Pf41 | Pf41 | gene synthesis, expressed as biotinylated proteins in MEK293E cells - full length | Julian Rayner,  Wellcome Trust Sanger Institute, UK |
| Circumsporozoite Protein | CSP | synthetic peptide: (PNAN) X    5 with N-terminal cystine coupled to BSA; mw 2.1kDa | AnaSpec, Inc. San Jose, CA |
| Liver-Stage Antigen-1 | LSA-1 | synthetic peptide: AKEKLQGQQSDLEQERLAKEKLQEQQSDLE QERLAKEKL | AnaSpec, Inc.  San Jose, CA |
| VAR2CSA (FcR3) | Fv2 | Baculovirus / Sf9; GU249598; amino acids M1 to F2649 | Ali Salanti, Univ of Copenhagen |
