# Supplementary material for: An analytical approach to reduce between-plate variation in multiplex assays that measure antibodies to Plasmodium falciparum antigens
Source: Malar J. 2017 Jul 17;16:287. doi: 10.1186/s12936-017-1933-6 (PMC5513105; doi:10.1186/s12936-017-1933-6)
Supplement: Supplementary file 2 — Additional file 2: Table S2. Summary of MFI values in linear scale for the pooled control samples across six plates. [file 12936_2017_1933_MOESM2_ESM.doc]

Supplementary Table 2. Summary of MFI values in linear scale for the pooled control samples across six plates

| Antigen | PC1 | PC2 | PC3 | PC4 | PC5 | NC-CM | NC-USA |
| --- | --- | --- | --- | --- | --- | --- | --- |
| AMA1 | 24516 (450) | 24850 (743) | 24597 (504) | 24303 (415) | 24226 (448) | 23663 (186) | 791 (43) |
| EBA175 | 24788 (638) | 24543 (629) | 24076 (342) | 23967 (461) | 21474 (970) | 18935 (726) | 668 (66) |
| MSP1 | 24664 (624) | 18789 (416) | 12395 (424) | 3465 (252) | 1200 (187) | 4886 (326) | 735 (69) |
| MSP2 | 25863 (1405) | 25664 (1498) | 24160 (570) | 13884 (827) | 25323 (1168) | 16880 (1248) | 1551 (119) |
| MSP3 | 14313 (699) | 10287 (609) | 5493 (498) | 14726 (761) | 6223 (483) | 5323 (590) | 1409 (181) |
| FV2 | 10933 (1389) | 17802 (2809) | 16239 (1548) | 6400 (1153) | 13247 (1678) | 3029 (951) | 946 (277) |
| MSP11 | 4806 (250) | 4621 (212) | 4184 (190) | 4147 (268) | 4079 (258) | 4346 (191) | 3965 (210) |
| Pf41 | 3865 (264) | 4287 (295) | 3824 (289) | 2243 (183) | 1215 (143) | 2352 (237) | 220 (26) |
| CSP | 1640 (150) | 1537 (193) | 877 (100) | 551 (71) | 1026 (103) | 950 (127) | 348 (50) |

The data were present in mean (SD).
